# Molecular phylogenetic data and seed coat anatomy resolve the generic position of some critical Chenopodioideae (Chenopodiaceae – Amaranthaceae) with reduced perianth segments

**DOI:** 10.3897/phytokeys.109.28956

**Published:** 2018-10-24

**Authors:** Alexander P. Sukhorukov, Maya V. Nilova, Anastasiya A. Krinitsina, Maxim A. Zaika, Andrey S. Erst, Kelly A. Shepherd

**Affiliations:** 1 Dept. of Higher Plants, Biological Faculty, Lomonosov Moscow State University, Leninskie Gory 1/12, 119234, Moscow, Russia Lomonosov Moscow State University Moscow Russia; 2 Laboratory Herbarium (NS), Central Siberian Botanical Garden, Russian Academy of Sciences, Zolotodolinskaya st. 101, Novosibirsk, 630090, Russia Central Siberian Botanical Garden, Russian Academy of Sciences Novosibirsk Russia; 3 Laboratory of Systematics and Phylogeny of Plants (TK), Tomsk State University, Tomsk 634050, Russia, Tomsk State University Tomsk Russia; 4 Western Australian Herbarium, Department of Biodiversity, Conservation & Attractions, 17 Dick Perry Avenue, Kensington, Western Australia, 6151, Australia Western Australian Herbarium Kensington Australia

**Keywords:** *
Blitum
*, Chenopodioideae, *
Chenopodium
*, *
Oxybasis
*, new genus, taxonomy

## Abstract

The former Chenopodiumsubgen.Blitum and the genus *Monolepis* (Chenopodioideae) are characterised in part by a reduced (0–4) number of perianth segments. According to recent molecular phylogenetic studies, these groups belong to the reinstated genera *Blitum* incl. *Monolepis* (tribe Anserineae) and *Oxybasis* (tribe Chenopodieae). However, key taxa such as *C.antarcticum*, *C.exsuccum*, *C.litwinowii*, C.foliosumsubsp.montanum and *Monolepisspathulata* were not included and so their phylogenetic position within the Chenopodioideae remained equivocal. These species and additional samples of *Blitumasiaticum* and *B.nuttallianum* were incorporated into an expanded phylogenetic study based on nrDNA (ITS region) and cpDNA (*trnL-trnF* and *atpB-rbcL* intergenic spacers and *rbcL* gene). Our analyses confirm the placement of *C.exsuccum*, *C.litwinowii* and C.foliosumsubsp.montanum within *Blitum* (currently recognised as *Blitumpetiolare*, *B.litwinowii* and B.virgatumsubsp.montanum, respectively); additionally, *C.antarcticum*, currently known as *Oxybasisantarctica*, is also placed within *Blitum* (reinstated here as *B.antarcticum*). Congruent with previous studies, two of the three accepted species of *Monolepis* – the type species *M.trifida* (= *M.nuttalliana*) as well as *M.asiatica* – are included in *Blitum*. The monotypic genus *Carocarpidium* described recently with the type *C.californicum* is not accepted as it is placed within *Blitum* (reinstated here as *B.californicum*). To date, few reliable morphological characters have been proposed that consistently distinguish *Blitum* (incl. two *Monolepis* species) from morphologically similar *Oxybasis*; however, two key differences are evident: (1) the presence of long-petiolate rosulate leaves in *Blitum* vs. their absence in *Oxybasis* and (2) a seed coat structure with the outer wall of the testa cells lacking stalactites (‘non-stalactite seed coat’) but with an obvious protoplast in *Blitum* vs. seed coat with the outer walls of the testa cells having stalactites (‘stalactite seed coat’) and a reduced protoplast in *Oxybasis*. Surprisingly, the newly sequenced North American *Monolepisspathulata* nested within the tribe Dysphanieae (based on ITS and *trnL-trnF* + *rbcL* + *atpB-rbcL* analyses).The phylogenetic results, as well as presence of the stalactites in the outer cell walls of the testa and lack of the rosulate leaves, confirm the distinctive nature of *Monolepisspathulata* from all *Blitum* and, therefore, the recent combination *Blitumspathulatum* cannot be accepted. Indeed, the morphological and molecular distinctive nature of this species from all Dysphanieae supports its recognition as a new monotypic genus, named herein as *Neomonolepis* (type species: *N.spathulata*). The basionym name *Monolepisspathulata* is also lectotypified on a specimen currently lodged at GH. Finally, while *Micromonolepispusilla* is confirmed as belonging to the tribe Chenopodieae, its position is not fully resolved. As this monotypic genus is morphologically divergent from *Chenopodium*, it is retained as distinct but it is acknowledged that further work is required to confirm its status.

## Introduction

The family Chenopodiaceae Vent. comprises ~1500 species distributed worldwide (Sukhorukov 2014). It is divided into several subfamilies and at least one third of them belong to the core subfamily Chenopodioideae in the tribes Axyrideae G.Kadereit & Sukhor. (*Axyris* L., *Ceratocarpus* L., *Krascheninnikovia* Gueldenst.), Chenopodieae incl. Atripliceae Duby (*Archiatriplex* G.L.Chu, *Atriplex* L., *Chenopodiastrum* S.Fuentes, Uotila & Borsch, *Chenopodium* L. s.str., *Exomis* Fenzl ex Moq., *Extriplex* E.H.Zacharias, *Grayia* Hook. & Arn., *Halimione* Aellen, *Holmbergia* Hicken, *Lipandra* Moq., *Manochlamys* Aellen, *Microgynoecium* Hook.f., *Micromonolepis* Ulbrich, *Oxybasis* Kar. & Kir., *Proatriplex* Stutz & G.L.Chu and *Stutzia* E.H.Zacharias), Anserineae (*Blitum* L. incl. *Scleroblitum* Ulbr., *Spinacia* L.) and Dysphanieae (*Cycloloma* Moq., *Dysphania* R.Br., *Suckleya* A.Gray and *Teloxys* Moq.) (Kadereit et al. 2003, 2010; Zacharias and Baldwin 2010; Fuentes-Bazan et al. 2012a, 2012b). While tribal boundaries are becoming well established, the status of a number of genera is far from stabilised, as ongoing molecular phylogenetic analyses continue to highlight new and sometimes unexpected relationships.

Some of the most recent and drastic taxonomic changes have been proposed by Fuentes-Bazan et al. (2012b) following their phylogenetic study of the large genus *Chenopodium* (~200–250 species) (Fuentes-Bazan et al. 2012a) and this classification is currently accepted by many authors (e.g. Iamonico 2011, 2014; Mosyakin 2013; Uotila 2017; Sukhorukov et al. 2013; Sukhorukov and Kushunina 2014, Hernández-Ledesma et al. 2015; Mosyakin and Iamonico 2017). According to the findings by Fuentes-Bazan et al. (2012b), *Chenopodiumsensu lato* was shown to be polyphyletic and members previously included in the genus are now placed in tribes Chenopodieae incl. Atripliceae (*Chenopodium* s.str. 100–150 spp., *Oxybasis* ~12 spp., *Chenopodiastrum* 8–9 spp., *Lipandra* Moq., 1 sp.), Dysphanieae (*Dysphania* >50 spp., *Teloxys* 1 sp.) and Anserineae (*Blitum* ~12 spp.). To accomplish this, they reinstated the genera *Oxybasis* (type species *O.minutiflora* Kar. & Kir. = *O.chenopodioides* (L.) S.Fuentes, Uotila & Borsch) and *Lipandra* (type species *L.polysperma* (L.) Moq. ≡ *Chenopodiumpolyspermum* L.) and recognised the new genus *Chenopodiastrum* S.Fuentes, Uotila & Borsch. Finally, two of three known species of the genus *Monolepis* Schrad. included in the study (the type species *M.trifida* (Trev.) Schrad. = *M.nuttalliana* (Schult.) Greene) as well as *M.asiatica* Fisch. & C.A.Mey.) were shown to be nested within *Blitum* based on ITS (nrDNA) and *trnF* intergenic spacer with moderate statistical support (Fuentes-Bazan et al. (2012a). As *Blitum* is the oldest available name (Linnaeus 1753), *Monolepisasiatica* was transferred and *M.nuttalliana* was re-instated as *Blitumasiaticum* (Fisch. & C.A.Mey.) Fuentes et al. and *B.nuttallianum* Schult., respectively (Fuentes-Bazan et al. 2012b). The third *Monolepis* species, *M.spathulata* A.Gray, was not sequenced, but also transferred into *Blitum* [as *B.spathulatum* (A.Gray) Fuentes et al.] due to its morphological similarity to both *B.asiaticum* and *B.nuttallianum*.

Further changes were subsequently proposed by Theodorova (2014), provided without a detailed explanation, suggesting that *Blitum* should be expanded to include *Lipandra*, *Oxybasis* and *Chenopodiastrum*, resulting in the proposed new combinations *Blitumurbicum* (L.) T.A.Theodorova (≡ *Oxybasisurbica* (L.) S.Fuentes, Uotila & Borsch), *B.polyspermum* (L.) T.A.Theodorova (≡ *Lipandrapolysperma* (L.) S.Fuentes, Uotila & Borsch) and *B.hybridum* (L.) T.A.Theodorova (≡ *Chenopodiastrumhybridum* (L.) S.Fuentes, Uotila & Borsch). Recently, Zhu and Sanderson (2017) described a new monotypic genus *Carocarpidium* S.C.Sanderson et C.L.Chu with the type species *C.californicum* (S.Wats.) S.C.Sanderson & G.L.Chu (≡ *Blitumcalifornicum* S.Wats. ≡ *Chenopodiumcalifornicum* (S.Wats.) S.Wats.), based on the fruits having a fleshy pericarp.

The recent split of *Chenopodiumsensu lato* into genera belonging to different tribes as suggested by Fuentes-Bazan et al. (2012b) is supported in part by morphological characters. First, all species of *Chenopodium* with obvious glandular hairs, ovoid or roundish, yellow or orange subsessile glands and simple hairs now belong to the tribe Dysphanieae (placed in either *Dysphania* R.Br. or *Teloxys* Moq.), while the remaining former *Chenopodium* (now included in Chenopodieae and Anserineae) have an indumentum of white bladder (“mealy”) hairs, sometimes with scattered simple hairs (Reimann and Breckle 1988; Simón 1997; Sukhorukov et al. 2015b). The number of perianth segments was also traditionally thought to be a good diagnostic character, which usually corresponds to the number of stamens. *Chenopodium* s.str., *Lipandra* and *Chenopodiastrum* are characterised by the presence of five perianth segments and five stamens, while various genera across the subfamily are characterised by a lower number (1–4) of perianth segments and stamens, as observed in some *Oxybasis* and *Micromonolepis* (Chenopodieae), *Blitum* incl. *Monolepis* (Anserineae) and many *Dysphania* (Dysphanieae), especially amongst Australian species (e.g. Ulbrich 1934; Wilson 1984; Judd and Ferguson 1999; Holmgren 2003). However, this character may not be consistently informative as species such as *Oxybasisurbica* usually has 5 perianth segments and 5 stamens.

It has become apparent in recent years that fruit and seed characters are also useful in distinguishing members of the former *Chenopodium*, particularly amongst groups that are quite morphologically similar (Sukhorukov 2006, 2014; Sukhorukov and Zhang 2013; Sukhorukov et al. 2015a). A good example is *Chenopodiumgubanovii* Sukhor. Originally this species was described as a member of the former Chenopodiumsubgen.Blitumsect.Pseudoblitum (Sukhorukov 1999). Its generic status was discussed by Fuentes-Bazan et al. (2012b) and finally resolved by Sukhorukov et al. (2013) as being a part of *Oxybasis* [*Oxybasisgubanovii* (Sukhor.) Sukhor. et Uotila] based on molecular phylogenetic data supported by morphological and seed characters. Almost all Chenopodieae (*Archiatriplex*, *Chenopodium*, *Chenopodiastrum*, *Exomis*, *Holmbergia*, *Lipandra*, *Manochlamys*, *Microgynoecium*, *Proatriplex* and all *Atriplex* with red or black seeds) possess a seed-coat testa with thickened outer cell walls impregnated with vertical or oblique stalactites and a reduced protoplast (hereafter ‘stalactite seed coat’) (Sukhorukov 2006; Kadereit et al. 2010; Sukhorukov and Zhang 2013; Sukhorukov 2014). There are a few exceptions, however, for example the seed coat in *Halimione* and three *Chenopodium* species endemic to Juan Fernández Archipelago (Chile) (*C.nesodendron* Skottsb., *C.sanctae-clarae* Johow, *C.sancti-ambrosii* Skottsb.), does not contain the stalactites in the outer cell walls and possesses a visible protoplast (hereafter ‘non-stalactite seed coat’) (Sukhorukov 2014). These three geographically isolated Chilean species are closely allied and highly unusual, as they not only possess a non-stalactite seed coat but have a tree-like habit and fruits with an apically swollen pericarp. Of these, only *C.sanctae-clarae* has been included in molecular analyses (Kadereit et al. 2010), which confirmed its phylogenetic position within this genus. The non-stalactite seed coat morphology is also evident in the Dysphanieae, *Chenopodiumantarcticum* Hook.f [≡ *Oxybasisantarctica* (Hook.f.) Mosyakin], almost all *Blitum* sensu Fuentes-Bazan et al. (2012b) with the exception of *Blitumspathulatum* (A.Gray) S.Fuentes, Uotila & Borsch, or *Monolepisspathulata* (Sukhorukov 2014).

Amongst the species of the former *Chenopodium* or *Monolepis* investigated carpologically but not included in recent molecular phylogenetic studies, two taxa are of special interest. The first, *Monolepisspathulata*, is endemic to western states of USA and North Mexico and was transferred to *Blitum* (as *B.spathulatum*) due to morphological affinities with other species of the genus. The second taxon, *Chenopodiumantarcticum*, is another poorly known taxon endemic to Tierra del Fuego (southernmost parts of Argentina and Chile) that still occupies a pending position within Chenopodioideae. Previously, it was described as *Blitumantarcticum* Hook.f. (Hooker 1847) and later transferred by the same author to *Chenopodium* as *C.antarcticum* (Hook.f.) Hook.f. (Bentham and Hooker 1880). The latter name was widely accepted in subsequent taxonomic treatments (Reiche 1911; Aellen 1929, 1931; Aellen and Just 1943; Moore 1983; Giusti 1984; Zuloaga and Morrone 1999). Recently, *Chenopodiumantarcticum* was transferred into *Oxybasis* by Mosyakin [2013, as *O.antarctica* (Hook.f.) Mosyakin] based on its morphological similarity to other *Oxybasis*. However, the stalactite seed coat morphology of *Blitumspathulatum* and non-stalactite seed coat of *Oxybasisantarctica* contrast with those of other members of *Blitum* and *Oxybasis*, respectively (Sukhorukov 2014), which raises the question of their true phylogenetic position.

To resolve this issue, we have included these two species, in addition to several accessions of taxa sampled for the first time [*Chenopodiumantarcticum*, *C.exsuccum* (C.Loscos) Uotila, *C.litwinowii* (Paulsen) Uotila, C.foliosum(Moench)Asch.subsp.montanum Uotila and *Monolepisspathulata*], as well as an additional sample of *Blitumasiaticum* (Fisch. & C.A.Mey.) S.Fuentes, Uotila & Borsch. in expanded molecular analyses based on nrDNA (ITS region) and cpDNA (*atpB-rbcL* intergenic spacers + *rbcL* and *trnL-trnF* intergenic spacer + *rbcL*, hereafter as *atpB-rbcL* and *trnL-trnF*, respectively) to determine their phylogenetic position within the Chenopodioideae. Furthermore, we discuss the role of fruit and seed characters for delimitating morphologically similar but phylogenetically distant taxa and conclude with proposed taxonomic changes that reflect our findings.

## Methods

### Taxon sampling

Several new taxa were included in the phylogenetic analysis for the first time: *Chenopodiumantarcticum* (Hook.f.) Hook.f. [≡ *Oxybasisantarctica* (Hook.f.) Mosyakin: Chile, Tierra del Fuego, December 1971, *Moore & Goodall s.n*. (LE)]; *C.exsuccum* (C.Loscos) Uotila: Algeria, Zenina, July 1968, *V.P. Boczantsev 681* (LE); C.foliosum(Moench)Asch.subsp.montanum Uotila: Iran, prov. Tehran, Elburz, June 1977, *K.-H. Rechinger 57243* (B); *C.litwinowii* (Paulsen) Uotila: Afghanistan, Parwan prov., Salang, 8 August 1969, *J.E. Carter 602* (LE); *Monolepisspathulata* A.Gray: USA, California, Susanville, August 1983, *I.Yu. Koropachinsky & al*. *404* as *Monolepisnuttalliana* (MHA). Additionally, we have included a new accession of *Blitumasiaticum* (Fisch. et C.A.Mey.) S.Fuentes, Uotila et Borsch (Russia, Yakutiya, Ust-Yansky distr., August 1976, *E.V. Ter-Grigoryan 1009*, MHA). The taxa included in the molecular analyses and their GenBank accession numbers are given in the Table 1.

**Table 1.** Voucher information and GenBank accession numbers for the species of Chenopodioideae and outgroups included in the phylogenetic analysis (arranged in alphabetical order). The newly sequenced samples are highlighted in bold. Some vouchers in GenBank may be stored under old names.

Species	Old names (if applicable)	GenBank accession number			
		ITS	*rbcL*	*trnL-trnF*	*atpB-rbcL*
* Atriplex hortensis *	–	HM005854	KX678160	HE577500	–
* Atriplex patula *	–	HE577358	MG249776	HE577498	HM587650
* Atriplex spongiosa *	–	–	AY270060	–	HM587661
* Atriplex undulata *	–	–	AY270061	–	HM587665
* Atriplex phyllostegia *	–	HM005870	HM587590	–	HM587651
* Atriplex peruviana *	–	HM005867	–	–	–
* Atriplex watsonii *	–	HM005871	–	–	–
* Atriplex rusbyi *	–	HM005865	–	–	–
* Atriplex patagonica *	–	HM587541	–	–	–
* Atriplex lentiformis *	–	HM005872	–	–	HM587637
* Atriplex cinerea *	–	HM587491	–	–	–
* Atriplex centralasiatica *	–	DQ086481	HM587583	–	HM587621
* Atriplex suberecta *	–	HM005863	–	–	–
* Axyris amaranthoides *	–	AM849227	KX678411	HE577510	–
* Axyris hybrida *	–	HE577371	–	HE577511	–
*** Blitum antarcticum ***	*Chenopodiumantarcticum* (*Oxybasisantarctica*)	**MH155315**	**MH632743**	**MH632745**	**MH152573**
*** Blitum asiaticum ***	* Monolepis asiatica *	**MH150882**	**MH731231**	**MH731229**	–
* Blitum bonus-henricus *	* Chenopodium bonus-henricus *	HE577372	KF613023	HE577512	HM587670
* Blitum californicum *	* Chenopodium californicum *	HE577376	MF963177	HE577516	–
* Blitum capitatum *	* Chenopodium capitatum *	KJ629064	MG249277	HE577513	–
*** Blitum litwinowii ***	* Chenopodium litwinowii *	**MH153781**	**MH632744**	**MH632746**	**MH632749**
* Blitum nuttallianum *	* Monolepis nuttalliana *	HE577375	JX848452	HE577515	HM587702
*** Blitum petiolare ***	* Chenopodium exsuccum *	**MH150883**	–	**MH632747**	**MH152574**
*Blitumvirgatum* L.	* Chenopodium foliosum *	JF976147	AY270081	HE577518	HM587673
** Blitum virgatum subsp. montanum **	Chenopodium foliosum subsp. montanum	**MH155242**	–	–	–
* Ceratocarpus arenarius *	–	AY556430	HM587594	HE577505	–
* Chenopodiastrum coronopus *	* Chenopodium coronopus *	HE577403	HM587595	HE577543	HM587671
* Chenopodiastrum hybridum *	* Chenopodium hybridum *	HE577530	–	HE577530	–
* Chenopodiastrum murale *	* Chenopodium murale *	HE577392	HM849890	HE577531	HM587675
* Chenopodium album *	–	JF976146	JF941270	HE577609	MF073794
* Chenopodium atrovirens *	–	KP226648 /	KX679232	HE577587	–
* Chenopodium auricomum *	–	KP226671	–	–	–
* Chenopodium bengalense *	* Chenopodium giganteum *	HE577458	–	–	–
Chenopodium berlandieri var. boscianum	–	HE577426	MG249740	HE577564	–
Chenopodium berlandieri var. zschackei	–	HE577425	–	–	–
* Chenopodium desertorum *	–	HE577417	AY270042	HE577555	HM587672
* Chenopodium desiccatum *	–	HE577412	KX678128	HE577550	–
* Chenopodium ficifolium *	–	HE577466	KM360714	HE577606	–
* Chenopodium fremontii *	–	HE577408	KX679065	HE577572	–
* Chenopodium hians *	–	HE577470	MG248000	HE577610	–
* Chenopodium iljinii *	–	HE577468	–	–	–
* Chenopodium incanum *	–	HE577410	MG246401	HE577548	–
* Chenopodium leptophyllum *	–	HE577428	MG248863	HE577566	–
* Chenopodium neomexicanum *	–	KJ629054	–	–	–
* Chenopodium nevadense *	–	HE577411	–	–	–
* Chenopodium opulifolium *	–	HE577454	MG248036	HE577594	–
* Chenopodium pallescens *	–	HE577409	–	–	–
* Chenopodium pallidicaule *	–	KJ629055	–	–	–
* Chenopodium nutans *	* Einadia nutans *	–	KM896090	–	HM587686
* Chenopodium parabolicum *	* Rhagodia parabolica *	–	KU564859	–	HM587704
* Chenopodium quinoa *	–	HE577443	KY419706	–	KY419706
* Chenopodium standleyanum *	–	KJ629051	MG249838	HE577560	–
* Chenopodium subglabrum *	–	HE577465	MG249459	HE577605	–
* Chenopodium vulvaria *	–	HE577407	JN892907	HE577591	–
* Chenopodium watsonii *	–	HE577462	MG246238	HE577602	–
* Cycloloma atriplicifolium *	–	HQ218998	HM587598	–	HM587681
* Dysphania ambrosioides *	* Chenopodium ambrosioides *	DQ005963	MG249540	HE577493	HM587682
* Dysphania botrys *	* Chenopodium botrys *	KJ629068	MG247946	DQ499383	HM587683
* Dysphania cristata *	* Chenopodium cristatum *	KJ629066	AY270046	–	HM587684
* Dysphania glomulifera *	* Chenopodium glomuliferum *	–	AY270086		HM587685
* Dysphania pumilio *	* Chenopodium pumilio *	HE577343	MG248652	HE577485	–
* Dysphania schraderiana *	* Chenopodium schraderianum *	HE577349	–	–	–
* Exomis microphylla *	–	–	HM587601	–	HM587687
* Grayia brandegeei *	–	HM005845	HM587604	HE577497	HM587690
* Grayia spinosa *	–	HM005844	HM587605	HE577496	HM587691
* Halimione verrucifera *	* Atriplex verrucifera *	HM587575	HM587606	–	HM587695
* Halimione pedunculata *	* Atriplex pedunculata *	HM587573	AY270093	–	HM587694
* Holmbergia tweedii *	–	HM005842	AY270100	–	HM587696
* Krascheninnikovia ceratoides *	–	HE577367	AY270105	HE577507	HM587697
Krascheninnikovia ceratoides subsp. lanata	* Krascheninnikovia lanata *	HE577368	MG248963	HE577508	HM587698
* Lipandra polysperma *	* Chenopodium polyspermum *	KJ629061	KX677934	HE855686	–
* Micromonolepis pusilla *	–	–	HM587608	–	HM587701
*** Neomonolepis spathulata ***	*Monolepisspathulata* (*Blitumspathulatum*)	**MH675518**	**MH731232**	**MH731230**	**MH152575**
* Oxybasis glauca *	* Chenopodium glaucum *	KJ629060	MG249300	HE577527	MF073807
* Oxybasis rubra *	* Chenopodium rubrum *	HE577381	MG249329	HE577525	–
* Oxybasis urbica *	* Chenopodium urbicum *	KJ629057	MG246691	HE577524	HM587678
* Oxybasis micrantha *	–	KU359325	–	–	–
* Spinacia oleracea *	–	EU606218	–	AJ400848	–
* Suckleya suckleyana *	–	HE577347	–	–	–
* Teloxys aristata *	*Chenopodiumaristatum*; *Dysphaniaaristata*	KJ629070	AY270140	–	HM587708
**Outgroups**	–				
* Bassia laniflora *	* Kochia laniflora *	KF785942	–	–	–
* Bassia prostrata *	* Kochia prostrata *	KF785963	AY270104	HE577478	KF785926
*Beta vulgaris*	–	AY858597	–	–	DQ074969
* Hablitzia tamnoides *	–	AY858590	AY270092	HE577475	JQ407841
* Polygonum aviculare *	–	–	MF158792	HQ843161	JN234937
Polygonum aviculare subsp. buxiforme	–	GQ339988	–	–	–

### DNA extraction

Total genomic DNA was extracted from herbarium samples according to Krinitsina et al. (2015). Following the homogenisation of plant fragments (MiniLys, Bertin Technologies, France), total DNA was extracted using the CTAB-method (Doyle and Doyle 1987) and further purified using AMPure Beads (Beckman Coulter, USA).

PCRs for two chloroplast markers (*atpB-rbcL* and *trnL-trnF*) and nrDNA (ITS region) were carried out in a Thermal Cycler T100 (Bio-Rad, USA) using primers and cycler programmes listed in Table 2. A 10 ng aliquot of DNA was used to make a 25 μl total volume reaction, containing 1 μM of each primer, 200 μM of each dNTP and 0.5 U Encyclo polymerases (Evrogen, Russia). PCR products were checked on 1.2% agarose gels and purified using AMPure Beads (Beckman Coulter, USA) according to the owner’s manual. AMPure Beads suspension was mixed with a solution containing PCR-product ratio 1 vol. PCR-mix: 1.2 vol. AMPure Beads for *atpB-rbcL* and ITS primer pairs and 1 vol. PCR-mix: 1.4 vol. AMPure Beads for *rbcL*, Tab C/Tab D and Tab E/Tab F primer pairs.

**Table 2.** Primers and cycler programmes used for the molecular analysis.

Marker	Primer sequences and combination	Reference	Cycler programmer
ITS	ITS5 5'-GGA AGT AAA AGT CGT AAC AAG G-3'	White et al. (1990)	95 °C for 5 min, 33 cycles of amplification (95 °C for 15 s, 55 °C for 30 s, 72 °C for 40 s), 72 °C for 5 min
	ITS4 5'-TCC TCC GCT TAT TGA TAT GC-3'		
*rbcL* (partial)	rbcLaF 5'- ATG TCA CCA CAA ACA GAG ACT AAA GC-3'	Levin et al. (2003)	95 °C for 5 min, 35 cycles of amplification (95 °C for 10 s, 55 °C for 30 s, 72 °C for 40 s), 72 °C for 5 min
	rbcLaR 5'-GTA AAA TCA AGT CCA CCR CG-3'	Kress et al. (2009)	
*atpB-rbcL* spacer	atpB-rbcL F 5'-GAA GTA GTA GGA TTG ATT CTC-3'	Golenberg et al. (1993)	95 °C for 5 min, 35 cycles of amplification (95 °C for 20 s, 56 °C for 30 s, 72 °C for 60 s), 95 °C for 20 s, 56 °C for 80 s, 72 °C for 8 min
	atpB-rbcL R 5'-CAA CAC TTG CTT TAG TCT CTG-3'		
*trnL-F*	Tab C 5'-CGA AAT CGG TAG ACG CTA CG-3'	Taberlet et al. (1991)	95 °C for 5 min, 35 cycles of amplification (95 C for 1 min, 50 °C – 65 °C (increasing in 0.3 C per cycle) for 1 min, 72 °C for 4 min), 72 °C for 5 min
	Tab D 5'-GGG GAT AGA GGG ACT TGA AC-3'		
	Tab E 5'- GGT TCA AGT CCC TCT ATC CCC-3'		
	Tab F 5'ATI' TGA ACT GGT GAC ACG AG 3'		

### Sequencing and alignment

Sequencing was performed following Sanger methods on an Applied Biosystems 3730 DNA Analyser using ABI PRISM BigDye Terminator v. 3.1 (Center of Collective Use “Genome”, Institute of Molecular Biology, Moscow, Russia). The sequencing primers were the same as the amplification primers.

The raw forward and reverse sequences were checked and combined in BioEdit sequence alignment editor v. 7.0.5.3 (Hall 1999). Sequences were edited and aligned using Muscle 3.6 (Edgar 2004). The obtained alignments were manually edited using PhyDe (version 0.9971: Müller et al. 2010) following the rules outlined in Löhne and Borsch (2005). Mutational hotspots (regions of uncertain homology) were excluded from the analysis (Borsch et al. 2003). Gaps were treated as missing data during the phylogenetic inference.

### Phylogenetic inference

To show the relationships between taxa, we reconstructed various phylogenies using Bayesian analysis, maximum likelihood (ML) and maximum parsimony (MP) methods for the ITS and combined *trnL-trnF* + *rbcL* + *atpB-rbcL* datasets. Models of nucleotide substitution were selected using the MrModeltest 2.1.7 (Nylander 2004) via the Akaike information criterion (AIC: Akaike 1974). The substitution model was set to GTR + G + I. For the ML analyses, we employed RAxML Version 8 (Stamatakis 2014). Bootstrap analyses were conducted with 2500 replicates for ML. Parsimony analyses were conducted in PAUP* 4.0a162 (Swofford 2002) with the following settings: all characters have equal weight, MaxTrees set to 1000 (auto increased by 1000), TBR branch swapping and with 20000 jackknife (JK) replicates to calculate node support. Bayesian analyses were conducted in BEAST 2.5.0 (Bouckaert et al. 2014). Four Markov Chain Monte Carlo analyses with four chains were run for 20 million generations for every dataset, sampling every 1000 generations. Burn-in was set to remove 5% of the total trees sampled after assessing likelihood convergence by inspection of the trace plots in the programme Tracer v.1.6 (Rambaut et al. 2014). A birth and death prior was chosen for branch lengths (Gernhard 2008). The maximum clade credibility tree was calculated in the programme TreeAnnotator v1.4.8 (Drummond and Rambaut 2007) with a posterior probability limit of 0.7. Final trees were edited in the programme TreeGraph ver. 2.14.0 (Stöver and Müller 2010).

### Morphology and anatomy

The carpology of the tribe Chenopodioideae was described in detail in a previous study by Sukhorukov (2014). In this study, we pay particular attention to the fruit and seed of *Chenopodiumantarcticum* and to the general structure of the reproductive shoot of *Monolepisspathulata* that were not illustrated in Sukhorukov (2014). The samples were observed using a scanning electron microscope (SEM) JSM–6380 (JEOL Ltd., Japan) at 15 kV after sputter coating with gold-palladium in the laboratory of Electron Microscopy at Lomonosov Moscow State University. Prior to SEM, the fruits were dehydrated in aqueous ethyl alcohol solutions of increasing concentration, followed by alcohol-acetone solutions and pure acetone. No dehydration of the seeds is required prior to SEM observation due to the absence of soft tissues (e.g. papillae or trichomes) on their surface.

The cross-sections of the seeds were prepared using a rotary microtome Microm HM 355S (Thermo Fisher Scientific, USA) and then examined using a Nikon Eclipse Ci (Nikon Corporation, Japan) light microscope and photographed using a Nikon DS-Vi1 camera (Nikon Corporation, Japan) at the Department of Higher Plants, Lomonosov Moscow State University. Before sectioning, the seeds were soaked in water:alcohol:glycerine (1:1:1) solution, dehydrated in ethanol dilution series and embedded in the Technovit 7100 resin (Heraeus Kulzer, Germany).

## Results

### Phylogenetic analysis

**Figure 1.**
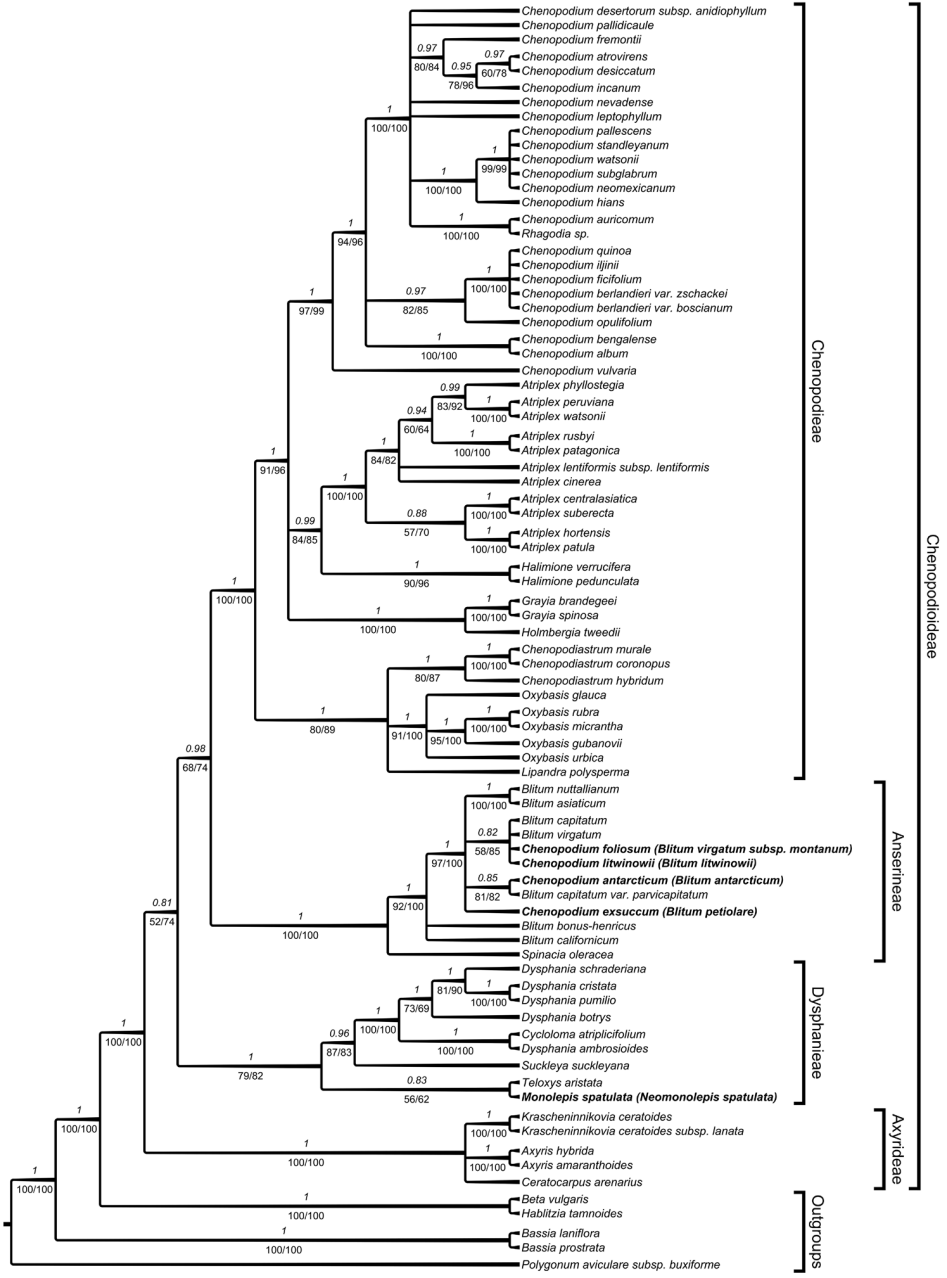
Best tree from the BEAST analysis of the ITS Chenopodioideae dataset. Bayesian posterior probabilities are given above the branches, jackknife values (left) and bootstrap percentages of the maximum likelihood analyses (right) are given below branches.

**Figure 2.**
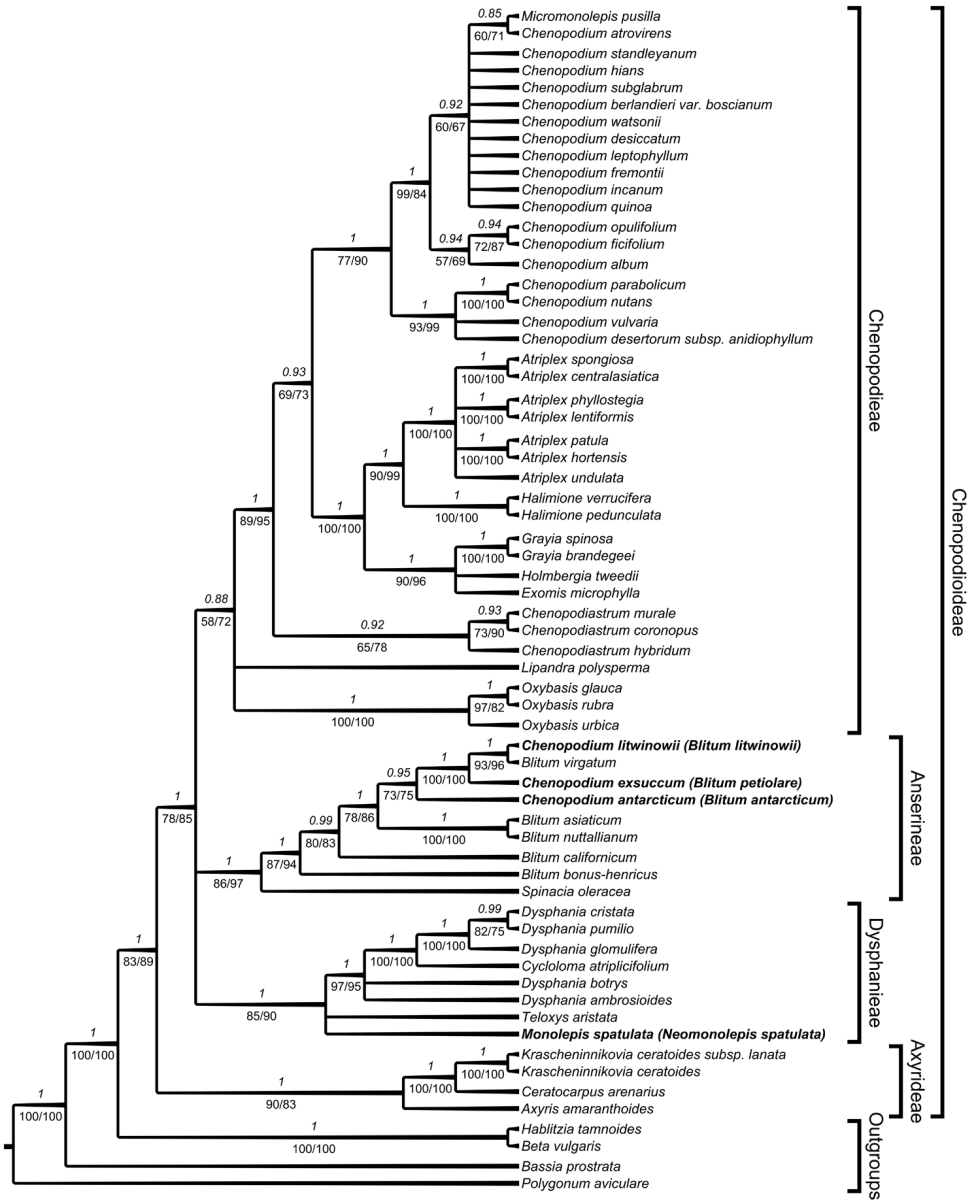
Best tree from the BEAST analysis of the combined *trnL-trnF* + *rbcL* + *atpB-rbcL*Chenopodioideae dataset. Bayesian posterior probabilities are given above the branches, jackknife values (left) and bootstrap percentages of the maximum likelihood analyses (right) are given below branches.

The phylogenetic analysis based on nrDNA (ITS) and combined cpDNA analyses (*trnL-trnF* + *rbcL* + *atpB-rbcL*) revealed that the tribes Axyrideae, Chenopodieae s.str., Anserineae and Dysphanieae are well-supported within Chenopodioideae and congruent with previous molecular analyses by Fuentes-Bazan et al. (2012b) (Figures 1–2). The results outlined below focus on the phylogenetic position of the newly included taxa *Chenopodiumantarcticum* [≡ *Oxybasisantarctica*], *C.litwinowii*, *C.exsuccum*, C.foliosumsubsp.montanum and *Monolepisspathulata*.

In the ITS analysis (Figure 1), the tribe Axyrideae is placed sister to the remaining Chenopodioideae. The next diverging lineage is a well-supported Dysphanieae, with *Monolepisspathulata* + *Teloxys* forming a sister lineage to the remaining representatives of the tribe. *Chenopodiumantarcticum*, *C.litwinowii*, *C.exsuccum* and C.foliosumsubsp.montanum fall well within *Blitum*, which is sister to a well-supported Chenopodieae. *Blitumcalifornicum* and *B.bonus-henricus* (L.) C.A.Mey. form part of the polytomy with the rest of the genus.

Like the ITS phylogenetic analysis, the combined *trnL-trnF* + *rbcL* + *atpB-rbcL* tree (Figure 2) shows the Axyrideae as an early branching lineage in Chenopodioideae, sister to a polytomy of Dysphanieae, Anserineae and Chenopodieae. Within the Dysphanieae, *Monolepisspathulata* and *Teloxys* form a polytomy with the remaining representatives of the tribe, which includes *Cycloloma* nested within *Dysphania*. *Chenopodiumantarcticum*, *C.litwinowii* and *C.exsuccum* are nested within *Blitum* (C.foliosumsubsp.montanum is not included in the combined tree). *Chenopodiumantarcticum* is sister to *Chenopodiumexsuccum* + *C.litwinowii* – *Blitumvirgatum*.

### Carpological studies

**Figure 3.**
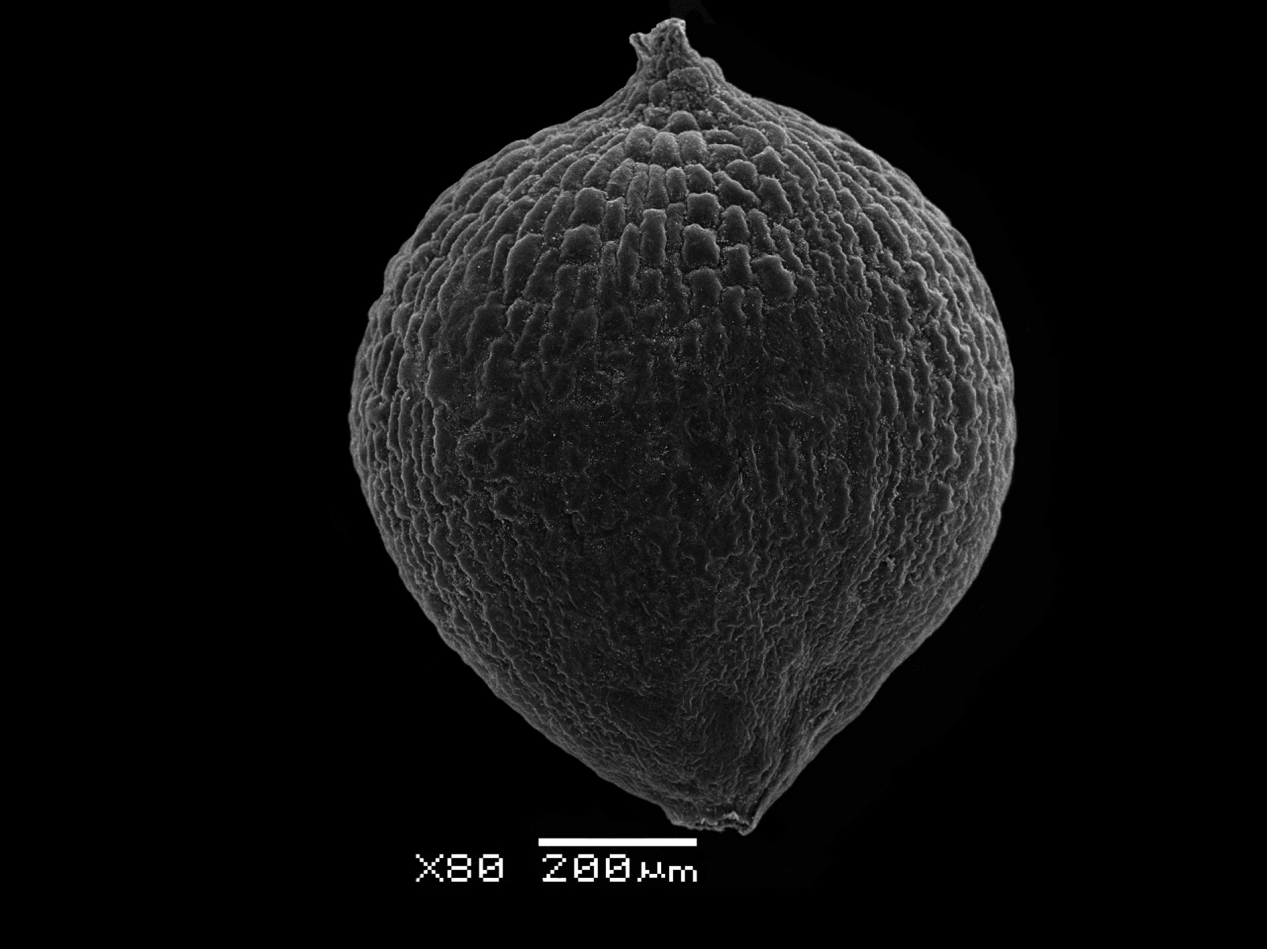
Pericarp of *Blitumantarcticum*. Scale bar: 200 μm.

**Figure 4.**
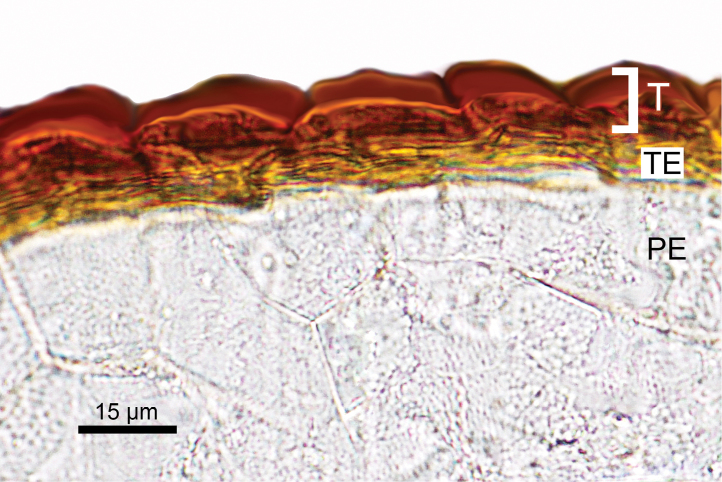
Cross-section of the seed of *Blitumantarcticum*. Abbreviations: T – testa, TE- tegmen, PE – perisperm.

**Figure 5.**
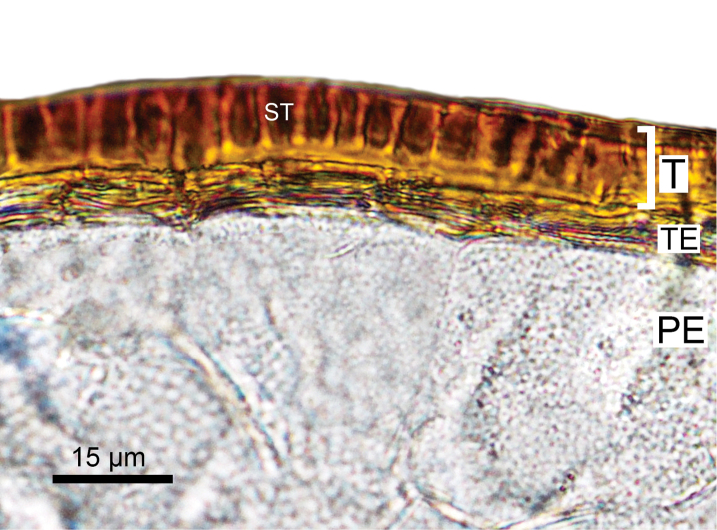
Cross-section of the seed of *Neomonolepisspathulata*. Abbreviations: T – testa, TE – tegmen, PE – perisperm, ST – stalactites in the outer walls of the testa cells.

This study highlighted the fact that these species, with the exception of *Monolepisspathulata*, possess the same fruit and seed anatomy as other *Blitum* species such as a mamillate pericarp (Figure 3) and non-stalactite seed-coat with obvious (visible) protoplast (Table 3; Figure 4). In contrast, the carpology of *Monolepisspathulata* somewhat resembles the morphology observed in species of *Oxybasis* and many other Chenopodieae in having a papillate pericarp and a stalactite seed coat with a highly reduced protoplast (Figure 5). Other important characters such as life history, the degree of fusion of reduced perianth segments, pericarp structure and adherence, the colour, shape and morphology of seeds and an embryo position, are recorded for representative species of each genus, as summarised in Table 3.

**Table 3.** Additional noteworthy characters evolved in *Blitum* and *Oxybasis*. This table summarises life history and carpological data from Sukhorukov and Zhang (2013), Sukhorukov et al. (2013), Sukhorukov (2014), with additional information included for Blitumvirgatumsubsp.montanum and *B.korshinskyi*.

Taxon/Character	Life history	Perianth segments	Cells of the outer pericarp layer	Pericarp adherence to the seed coat	Seed shape and colour	Seed surface	Seed keel	Thickness of seed-coat testa (µm)	Acicular outgrowths of the testa cells	Presence of spatial heterospermy	Seed embryo position
*** Blitum antarcticum ***	short-lived perennial herb	basally connate	spongy	scraped off the seed	roundish, red	alveolate	–	12–20	–	–	vertical
*** B. asiaticum ***	annual	free	not spongy	easily ruptured	roundish, red	undulate	+	7–10	–	–	vertical
*** B. atriplicinum ***	annual or short-lived perennial herb	basally connate	not spongy	hardly removed	roundish, red	alveolate, with hairy-like outgrowths	–	17–25	+	–	vertical
*** B. bonus-henricus ***	perennial herb	basally connate	spongy	scraped off the seed	roundish, red	smooth	–	37–45	–	+	vertical, rarely horizontal
*** B. californicum ***	perennial herb	basally connate	spongy	scraped off the seed	roundish, red	alveolate	–	25–30 and 37–45 (heterospermous)	–	+	vertical
*** B. capitatum ***	annual or short-lived perennial herb	basally connate	not spongy	hardly removed	ovate, red	undulate	+ (two keels and a groove between them)	12–15	–	+	vertical
*** B. hastatum ***	annual or short-lived perennial herb	connate to 1/3	not spongy	hardly removed	ovate, red	undulate	+ (two keels and a groove between them	15–18	–	+	vertical
*** B. korshinskyi ***	annual or short-lived perennial herb	almost free	not spongy	hardly removed	ovate, red	undulate	+ (two keels and a groove between them)	10–12	–	–	vertical
*** B. litwinowii ***	annual or short-lived perennial herb	basally connate	not spongy	hardly removed	ovate, red	alveolate	+ (two keels and a groove between them)	10–12	–	–	vertical
*** B. nuttallianum ***	annual	free, or perianth absent	not spongy	hardly removed	roundish, red	alveolate, with hairy-like outgrowths	–	8–10	+	–	vertical
*** B. petiolare ***	annual or short-lived perennial herb	basally connate	not spongy	hardly removed	ovate, red	alveolate	+ (two keels and a groove between them)	15–17	–	–	vertical
*** B. virgatum ***	annual or short-lived perennial herb	basally connate	not spongy	hardly removed	ovate, red	undulate	+ (two keels and a groove between them)	10–12	–	+	vertical
*** Oxybasis chenopodioides ***	annual	fused in almost all flowers, free only in some flowers	not spongy	easily ruptured	roundish, red	minutely pitted	–	10–15	+		vertical and horizontal
*** O. glauca ***	annual	basally connate	not spongy	easily ruptured	roundish, red	minutely pitted	–	10–15 and 17–25 (heterospermous)	+	+	vertical and horizontal
*** O. gubanovii ***	annual	basally connate	not spongy	hardly removed	roundish, red	smooth (minutely pitted)	+ (one keel)	12–15	+	–	vertical
*** O. macrosperma ***	annual	connate to the middle or almost to the top	spongy	scraped off the seed	roundish, red	reticulate with minutely pitted dots	–	12–20	+	–	vertical and horizontal
*** O. mexicana ***	annual	basally connate	not spongy	easily ruptured	roundish, red	reticulate with minutely pitted dots	–	20–25	+	+	vertical and horizontal
*** O. micrantha ***	annual	basally connate	not spongy	scraped off the seed	roundish, red	minutely pitted	+ (one keel)	12–15	+	–	horizontal, rarely vertical
*** O. rubra ***	annual	basally connate	not spongy	easily ruptured	roundish, red	reticulate with minutely pitted dots	–	10–15	+		vertical and horizontal
*** O. urbica ***	annual	basally connate	papillate	scraped off the seed	roundish, black	minutely pitted	–	42–50	+	–	horizontal

## Discussion

The phylogenetic position of Chenopodiumfoliosumsubsp.montanum [≡ BlitumvirgatumL.subsp.montanum (Uotila) S.Fuentes, Uotila et Borsch], *C.exsuccum* [= *Blitumpetiolare* Link] and *C.litwinowii* [≡ *B.litwinowii* S.Fuentes, Uotila et Borsch] within *Blitum* as proposed by Fuentes-Bazan et al. (2012b) was supported by the findings of this study. Indeed, the results were predictable due to the shared morphological and carpological affinities of these species to *B.virgatum*, such as the presence of a leaf rosette, tight adherence of the pericarp to the seed coat and the ovoid and keeled seeds having the same anatomical structure (e.g. Uotila 1993, 1997; Sukhorukov 2014). For this reason, while *Chenopodiumkorshinskyi* (Litv.) Minkw. has not been included in any molecular phylogenies to date, it should be treated as *Blitumkorshinskyi* Litv. (Fuentes-Bazan et al. 2012b) due to the shared presence of these diagnostic traits. It is also evident, based on phylogenetic and carpological data from this study, that *Oxybasisantarctica* (formerly *Chenopodiumantarcticum*) must be treated as *Blitumantarcticum* as proposed by Hooker (1847). Moreover, as *Oxybasisantarctica* is the type of Oxybasissect.Thellungia (Aellen) Mosyakin [including *Oxybasisantarctica* and *O.erosa* (R.Br.) Mosyakin: Mosyakin 2013], this section may be recognised within *Blitum* but this requires further exploration as the phylogenetic position of *B.antarcticum* remains equivocal.

### Diagnostic characters for *Blitum* and *Oxybasis*

**Figure 6.**
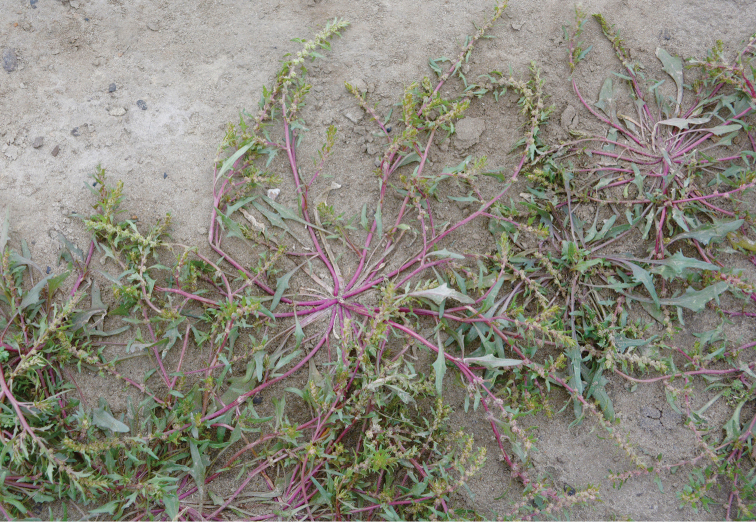
Habit of *Blitumasiaticum* showing the senescing leaf rosette. Photographer: Igor Pospelov (Russia, Krasnoyarsk prov., Taymyr, Khatanga, August 2014).

The importance of morphological characters used to delineate species within the genus *Chenopodium* that are now considered to belong to either *Blitum* or *Oxybasis* have been discussed by various authors (e.g. Moquin-Tandon 1840, 1849; Aellen and Just 1943; Scott 1978; Fuentes-Bazan et al. 2012b). However, the morphological similarity of some species has led to taxonomic confusion. For example, many macromorphological characters overlap in *Blitum* and *Oxybasis*, including previous diagnostic traits such as: reduced (1–4) number of perianth segments, presence of the vertical seed embryo position and emergence of spatial heterospermy. Such characters are clearly homoplastic in Chenopodieae, Anserineae and some other groups of the Chenopodioideae (Sukhorukov and Zhang 2013). Only one trait visible to the naked eye, the presence of leaf rosette in *Blitum* (Figure 6) and its absence in *Oxybasis*, can be used for the delimitation of both genera (see diagnostic key and generic descriptions in Fuentes-Bazan et al. 2012b). However, it should be noted that the leaf rosette in some *Blitum*, especially in species previously included in *Monolepis* (*B.asiaticum*, *B.nuttallianum*), is reduced to 1–2 leaves that may wither away completely by anthesis. From this study and from previous work (Sukhorukov and Zhang 2013; Sukhorukov et al. 2013; Sukhorukov 2014), it is evident that another character, the structure of the testa cells of the seed coat, is also diagnostic. In *Oxybasis*, as well as almost all other Chenopodieae, the seed testa cells have a reduced protoplast and “stalactites” hanging vertically in the outer wall (stalactite seed coat). In contrast, the presence of non-stalactite seed coat with a highly visible protoplast, unambiguously distinguishes *Blitum*. Other characters, such as reduced perianth segments, mamillate pericarp, red seeds, seed keel, vertical embryo position of note for representative species of each genus, are summarised in Table 3 and they play a role for the diagnostics at the species level or species group (see Sukhorukov 2014 for further detail).

In the absence of molecular phylogenetic data, it is clear that carpological characters must be taken into consideration when determining the generic placement of taxa in either *Blitum* or *Oxybasis*. Molecular data from this study and previous investigations (Kadereit et al. 2010; Fuentes-Bazan et al. 2012a, 2012b), when examined in conjunction with carpological evidence (Sukhorukov 2014), show that two taxonomic changes recently proposed: (1) the merger of *Oxybasis*, *Lipandra* and *Chenopodiastrum* (Chenopodieae) into an extended *Blitum* (Anserineae) as suggested by Theodorova (2014) and (2) the description of a new monotypic genus *Carocarpidium* S.C.Sanderson et G.L.Chu with the type *C.californicum* (≡*Blitumcalifornicum*) by Zhu and Sanderson (2017), cannot be accepted. Additionally, it should be noted that the pericarp of *B.californicum* is not fleshy as previously described (Zhu and Sanderson 2017), but its outer layer consists of spongy (mamillate) cells that imitate a “fleshy” pericarp. This type of mamillate pericarp is present in some *Blitum* and *Oxybasis* (Figure 3, see also Table 3) and so this character is clearly not unique to *Carocarpidium*.

### *Micromonolepis pusilla*

**Figure 7.**
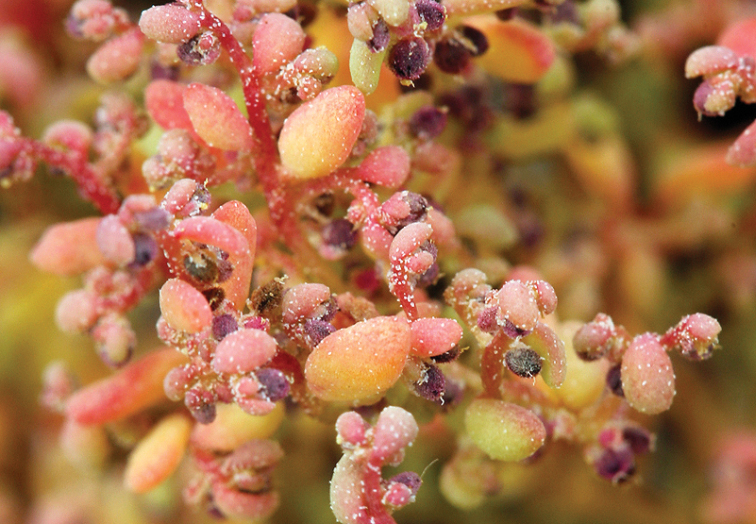
Shoot of *Micromonolepispusilla* showing the characteristic fleshy leaves. Photographer: Steve Matson (USA, California, Mono County, Long Valley, 2007).

This species was initially described as *Monolepispusilla* Torr. ex Watson (Watson 1871) and it is noteworthy to consider its morphology and phylogenetic position in context with other species previously known as *Monolepis*. It is a small annual herb covered with bladder hairs that has fleshy leaves (Figure 7), unisexual flowers with reduced (1–3) perianth segments and tiny papillate fruits. Due to its unusual habit, *M.pusilla* was transferred into a new monotypic genus *Micromonolepis* (Ulbrich 1934). The species was included in a *atpB-rbcL* molecular analysis, where it was unexpectedly placed within the “Chenopodieae I” clade comprising *Rhagodia*, *Einadia* and a part of *Chenopodium* s.l. (Kadereit et al. 2010). The papillate pericarp and the stalactite seed coat provide a good support for its placement into Chenopodieae, based on cpDNA being a part of *Chenopodium* s.str. (Kadereit et al. 2010, as Chenopodieae I; Figure 2). However, the limited number of taxa used in the *atpB-rbcL* analysis, the lack of additional molecular data and the significant morphological differences evident between *Micromonolepis* and the remaining *Chenopodium* species in this clade, such as the presence of fleshy leaves and reduced perianth segments, precludes the formal transfer of *M.pusilla* to *Chenopodium*. Further work is needed to evaluate the exact position of *Micromonolepispusilla* within Chenopodieae.

### *Monolepisspathulata* is neither *Monolepis* nor *Blitum*

Recently, *Monolepisspatulata* was transferred to *Blitum* (as *B.spathulatum*) based on its resemblance to other species of the genus due to the presence of a reduced number of perianth segments (Fuentes-Bazan et al. 2012b). It is evident, however, that the reduced number of perianth segments independently evolved in Chenopodieae (e.g. in *Micromonolepis* and some *Oxybasis*), Anserineae and many Dysphanieae (Sukhorukov and Zhang 2013). In light of carpological evidence (Sukhorukov 2014), it seemed doubtful that *M.spathulata* should be included in *Blitum*, as this species possesses a stalactite seed coat with a reduced protoplast. Our phylogenetic results show that *Monolepisspathulata* is not closely related to the other species in *Monolepis* (*M.asiatica*, and *M.nuttalliana*) that are now included in *Blitum* (Anserineae) as *B.asiaticum* and *B.nuttallianum*, respectively. This species falls within Dysphanieae forming a polytomy with *Teloxys* and *Dysphania* + *Cycloloma*. *M.spathulata* is a glabrous annual and differs from all Dysphanieae by the absence of simple hairs and subsessile glands that are diagnostic characters of this tribe. Additionally, *M.spathulata* is found to have the stalactite seed coat, a character missing in all Dysphanieae (Sukhorukov 2014). The close relationship between *M.spathulata* and the Dysphanieae, evidenced by molecular data, is unexpected given the obvious morphological and carpological differences. Indeed, *M.spathulata* is considered so distinct that it warrants recognition at the generic level. As the type for *Monolepis*, *M.trifida* (Trev.) Schrad. [= *M.nuttalliana* (Schult.) Greene], is synonymised within *Blitum* (as *Blitumnuttallianum*), a new name is required for *Monolepisspathulata*. As such, a new monotypic genus named *Neomonolepis* Sukhor., gen. nov. is established here.

## Taxonomy

### Neomonolepis

Taxon classificationPlantaeCaryophyllalesAmaranthaceae

Sukhor. gen. nov.

urn:lsid:ipni.org:names:77191294-1

#### Type species.

*Neomonolepisspathulata* (A.Gray) Sukhor., comb. nov.

#### Description.

**Figure 8.**
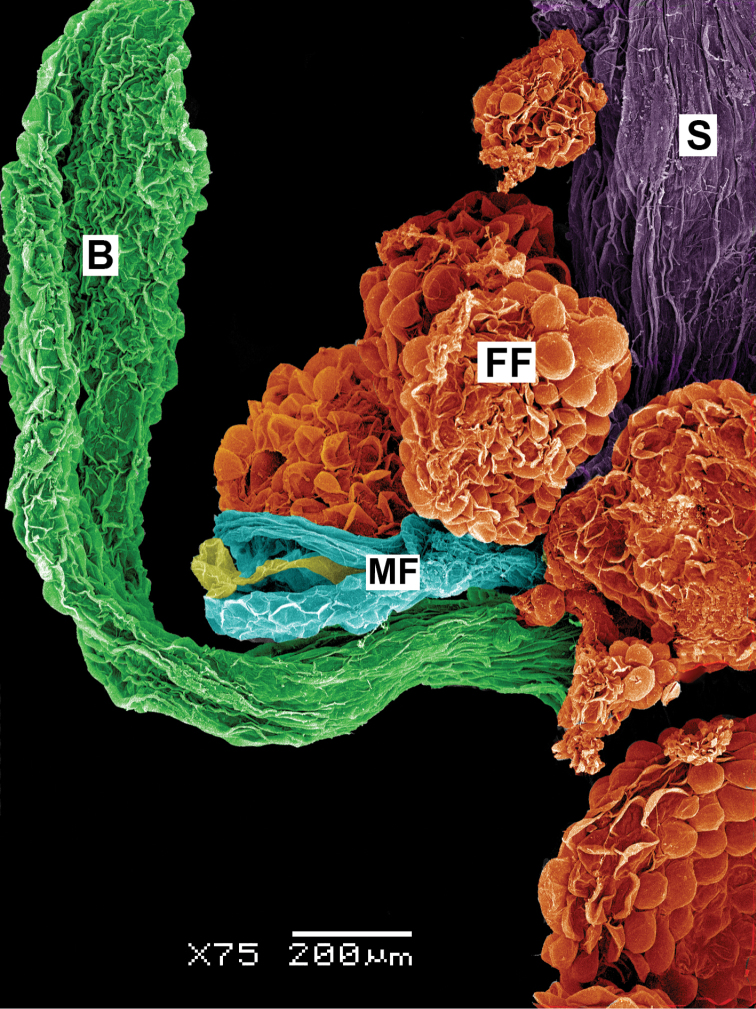
SEM detail of the inflorescence of *Neomonolepisspathulata*. Abbreviations: B – bract (stained in green), FF – female flowers (orange), MF –male flower (perianth stained in blue, stamen in yellow), S – stem.

Annual, glabrous, branched or not; lateral branches if present ascending; leaves cauline (rosulate leaves absent), densely located, spatulate-oblong, with a short petiole up to 1 cm or sessile, entire; inflorescence leafy (bracts similar to stem leaves); flowers sessile or shortly pedicellate, unisexual intermixed in small glomerules (Figure 8); male flowers with 2-lobed hyaline perianth, stamens 1–2, anthers 0.10–0.15 mm long; female flowers without perianth, fruits 0.55–0.65 mm in diameter, almost round, with blackish papillate pericarp (when dry) that is easily raptured, styles 2(3); seeds 0.4 × 0.3 mm, reddish, with smooth surface, with small irregular pits (seen at a higher magnification), seed-coat testa with stalactites in the outer cell walls and reduced protoplast; embryo vertical.

### Neomonolepis spathulata

Taxon classificationPlantaeCaryophyllalesAmaranthaceae

(A.Gray) Sukhor. comb. nov.

urn:lsid:ipni.org:names:77191295-1

≡ Monolepisspathulata A.Gray, Proc. Amer. Acad. Arts 7: 389 (1868). Lectotype (Sukhorukov, designated here): [USA, California, Sierra Nevada], Mono Pass, 1866, *H.N. Bolander 6373* lower right-hand specimen (GH00037208 [image]!, isolectotypes MO-216255 [image]! NY01085540 [image]! US00921387 [image]! YU064591 [image]!).
≡ Blitumspathulatum (A.Gray) S.Fuentes, Uotila et Borsch, Willdenowia 42(1): 17 (2012).

#### Morphological notes.

As *Neomonolepis* is a monotypic genus, the description of *N.spathulata* corresponds to the generic description above. *Neomonolepisspathulata* is morphologically distant from all Dysphanieae (*Teloxys*, *Suckleya* A.Gray, *Dysphania* R.Br. and *Cycloloma* Moq.) in being glabrous in all parts (vs. glandular and/or simple hairs), having unisexual flowers (vs. bisexual or polygamous) and ‘stalactite’ seed-coat testa (vs. ‘non-stalactite’). For this reason, we prefer to refer to the clade with the above-mentioned genera as the ‘Dysphanieae + Neomonolepis’ clade.

#### Typification.

The type specimen lodged at GH contains several plants collected from different areas in California and almost all of them were collected after the description of *Monolepisspathulata* (Gray 1868). The lectotype selected here (lower right-hand specimen on the GH00037208 sheet) is a part of original material cited in the protologue as “Sierra Nevada, at Mono Pass, in loose soil, Bolander” (Gray 1868) and it is chosen in accordance with Art. 9 of ICN (Turland et al. 2018). The description of the species is consistent with the image of the lectotype. Gray (1868) also noted that the seeds of *Monolepisspathulata* are notably smaller than those of *M.chenopodioides* [= *Blitumnuttallianum*]. The small seed dimensions of *Neomonolepisspathulata* (0.4 × 0.3 mm) are similar to those observed in many Australian *Dysphania* (Wilson 1984 sub *Chenopodium*; Sukhorukov 2014).

#### Distribution.

South-western North America (USA, North Mexico).

#### Etymology.

The new generic name is composed by the prefix “neo” (new) and the core name *Monolepis*.

## Conclusion

In the Chenopodioideae, some phylogenetically distant taxa often look similar due to convergence of various morphological characters, some of which were previously thought to be diagnostic such as the number of perianth segments. A remarkable example is highlighted by the different phylogenetic positions occupied by members of the former genus *Monolepis*, which are currently included in Anserineae (*M.nuttalliana* ≡ *Blitumnuttallianum*; *M.asiatica* ≡ *B.asiaticum*), Dysphanieae (*Neomonolepisspathulata* ≡ *Monolepisspathulata*) and Chenopodieae (*Monolepispusilla* ≡ *Micromonolepispusilla*). This study shows that fruit and seed characters such as seed-coat structure are valuable traits for taxonomic study. These features are particularly useful in distinguishing the morphologically similar but phylogenetically distinct genera *Blitum* and *Oxybasis*.

## Supplementary Material

XML Treatment for Neomonolepis

XML Treatment for Neomonolepis spathulata

## References

[B1] Aellen P (1929) Beitrag zur Systematik der *Chenopodium*-Arten Amerikas, vorwiegend auf Grund der Sammlung des United States National Museum in Washington I. Feddes Repertorium 26(1–6): 31–64. 10.1002/fedr.19290260108

[B2] Aellen P (1931) Die wolladventiven Chenopodien Europas. Verhandlungen der Naturforschenden Gesellschaft in Basel 41: 77–104.

[B3] Aellen P Just T (1943) Key and synopsis of the American species of the genus *Chenopodium* L. American Midland Naturalist 30(10): 47–76. 10.2307/2421263

[B4] Akaike H (1974) A new look at the statistical model identification. IEEE Transactions on Automatic Control 19(6): 716–723. 10.1109/TAC.1974.1100705

[B5] Bentham G Hooker JD (1880) Genera Plantarum, Vol. 3, part 1. Reeve & Co., London.

[B6] Borsch T Hilu KW Quandt D Wilde V Neinhuis C Barthlott W (2003) Noncoding plastid trnT-trnF sequences reveal a well resolved phylogeny of basal angiosperms. Journal of Evolutionary Biology 16(4): 558–576. 10.1046/j.1420-9101.2003.00577.x 14632220

[B7] Bouckaert R Heled J Kühnert D Vaughan T Wu CH Xie D Suchard MA Rambaut A Drummond AJ (2014) BEAST 2: A Software platform for Bayesian evolutionary analysis. PLoS Computational Biology 10(4): e1003537. 10.1371/journal.pcbi.1003537 PMC3985171 24722319

[B8] Doyle JJ Doyle JL (1987) A rapid DNA isolation procedure for small quantities of fresh leaf tissue. Phytochemical Bulletin 19: 11–15.

[B9] Drummond AJ Rambaut A (2007) BEAST: Bayesian evolutionary analysis by sampling trees. BMC Evolutionary Biology 7(1): 214. 10.1186/1471-2148-7-214 PMC2247476 17996036

[B10] Edgar RC (2004) MUSCLE: Multiple sequence alignment with high accuracy and high throughput. Nucleic Acids Research 32(5): 1792–1797. 10.1093/nar/gkh340 15034147 PMC390337

[B11] Flores-Olvera H Vrijdaghs A Ochoterena H Smets E (2011) The need to re-investigate the nature of homoplastic characters: An ontogenetic case study of the ‘bracteoles’ in Atripliceae (Chenopodiaceae). Annals of Botany 108(5): 847–865. 10.1093/aob/mcr203 21852278 PMC3177680

[B12] Fuentes-Bazan S Mansion G Borsch T (2012a) Towards a species level tree of the globally diverse genus *Chenopodium*. Molecular Phylogenetics and Evolution 62(1): 359–374. 10.1016/j.ympev.2011.10.006 22051350

[B13] Fuentes-Bazan S Uotila P Borsch T (2012b) A novel phylogeny-based generic classification for *Chenopodium* sensu lato, and a tribal rearrangement of Chenopodioideae (Chenopodiaceae). Willdenowia 42(1): 5–24. 10.3372/wi.42.42101

[B14] Gernhard T (2008) The conditioned reconstructed process. Journal of Theoretical Biology 253(4): 769–778. 10.1016/j.jtbi.2008.04.005 18538793

[B15] Giusti L (1984) Chenopodiaceae. In: Correa MN (Ed.) Flora Patagonica 4a, Tyrenc, Buenos Aires, 99–137.

[B16] Golenberg EM Clegg MT Durbin M Doebley J Ma DP (1993) Evolution of a noncoding region of the chloroplast genome. Molecular Phylogenetics and Evolution 2: 52–64. 10.1006/mpev.1993.1006 8081547

[B17] Gray A (1868) Characters of new plants of California and elsewhere, principally of those collected by H.N. Bolander in the State Geological Survey. Proceedings of the American Academy of Arts and Sciences 7: 327–401. 10.2307/20179569

[B18] Hall TA (1999) BioEdit: A user-friendly biological sequence alignment editor and analysis program for Windows 95/98/NT. Nucleic Acids Symposium Series 41: 95–98.

[B19] Hiitonen I (1933) Suomen Kasvio. Kustannsosakeyhtiö, Helsinki.

[B20] Hernández-Ledesma P Berendsohn WG Borsch T von Mering S Akhani H Arias S Castañeda-Noa I Eggli U Eriksson R Flores-Olvera H Fuentes-Bazán S Kadereit G Klak C Korotkova N Nyffeler R Ocampo G Ochoterena H Oxelman B Rabeler RK Sanchez A Schlumpberger BO Uotila P (2015) A taxonomic backbone for the global synthesis of species diversity in the angiosperm order Caryophyllales. Willdenowia 45(3): 281–383. 10.3372/wi.45.45301

[B21] Holmgren NH (2003) Gen. *Monolepis*, *Micromonolepis*. Flora of North America, North of Mexico, Vol. 4. Oxford University Press, New York & Oxford, 300–302.

[B22] Hooker JD (1847) The Botany of the Antarctic Voyage Vol. II: Flora Antarctica. Reeve & Co., London.

[B23] Iamonico D (2011) *Dysphaniaanthelmintica* (Amaranthaceae), new to the non-native flora of Italy, and taxonomic considerations on the related species. Hacquetia 10(1): 41–48. 10.2478/v10028-011-0002-x

[B24] Iamonico D (2014) Taxonomical, morphological, ecological and chorological notes on *Oxybasischenopodioides* and *O.rubra* (Chenopodiaceae) in Italy. Hacquetia 13(2): 297–302. 10.2478/hacq-2014-0005

[B25] Judd WS Ferguson IK (1999) The genera of Chenopodiaceae in the Southeastern United States. Harvard Papers in Botany 4(2): 365–416.

[B26] Kadereit G Borsch T Weising K Freitag H (2003) Phylogeny of Amaranthaceae and Chenopodiaceae and the evolution of C4 photosynthesis. International Journal of Plant Sciences 164(6): 959–986. 10.1086/378649

[B27] Kadereit G Mavrodiev EV Zacharias EH Sukhorukov AP (2010) Molecular phylogeny of Atripliceae (Chenopodioideae, Chenopodiaceae): Implications for systematics, biogeography, flower and fruit evolution, and the origin of C_4_ photosynthesis. American Journal of Botany 97(10): 1664–1687. 10.3732/ajb.1000169 21616801

[B28] Kress WJ Erickson DL Jones FA Swenson NG Perez R Sanjur O Bermingham E (2009) Plant DNA barcodes and a community phylogeny of a tropical forest dynamics plot in Panama. Proceedings of the National Academy of Sciences of the United States of America 106(44): 18621–18626. 10.1073/pnas.0909820106 19841276 PMC2763884

[B29] Krinitsina AA Zaika MA Speranskaya AS Sukhorukov AP Sizova TV (2015) A rapid and cost-effective method for DNA extraction from archival herbarium specimens. Biochemistry (Moscow) 80(11): 1478–1484. 10.1134/S0006297915110097 26615439

[B30] Ledebour CF (1829) . Flora Altaica, Vol. 1. Reimer, Berlin.

[B31] Levin RA Wagner WL Hoch PC Nepokroeff M Pires JC Zimmer EA Sytsma KJ (2003) Family level relationships of Onagraceae based on chloroplast *rbcL* and *ndhF* data. American Journal of Botany 90(1): 107–115. 10.3732/ajb.90.1.107 21659085

[B32] Linneaus C (1753) Species Plantarum, Vol. 1. Impensis Laurentii Salvii, Holmiae.

[B33] Löhne C Borsch T (2005) Molecular evolution and phylogenetic utility of the petD group II intron: A case study in basal angiosperms. Molecular Biology and Evolution 22(2): 317–332. 10.1093/molbev/msi019 15496557

[B34] Moore DM (1983) Flora of Tierra del Fuego. Missouri Botanical Garden, St. Louis.

[B35] Moquin-Tandon A (1840) Chenopodearum monographica enumeratio. Loss, Paris. 10.5962/bhl.title.15484

[B36] Moquin-Tandon A (1849) Salsolaceae [Chenopodiaceae]. In de Candolle A (Ed.) Prodromus systematis naturalis regni vegetabilis, Vol. 13(2). Typ. Masson, Paris, 43–219.

[B37] Mosyakin SL (1996) *Chenopodium* [s.l.]. In: Tzvelev NN (Ed.) Flora of Eastern Europe, Vol.9. Mir & Semya-95, St.-Petersburg, 27–44.

[B38] Mosyakin SL (2013) New nomenclatural combinations in *Blitum*, *Oxybasis*, *Chenopodiastrum*, and *Lipandra* (Chenopodiaceae). Phytoneuron 2013–56: 1–8.

[B39] Mosyakin SL Iamonico D (2017) Nomenclatural changes in *Chenopodium* (incl. Rhagodia) (Chenopodiaceae), with considerations on relationships of some Australian taxa and their possible Eurasian relatives. Nuytsia 28: 255–271.

[B40] Müller J Müller K Neinhuis C Quandt D (2010) PhyDE: Phylogenetic Data Editor v 0.9971. www.phyde.de

[B41] Nylander JAA (2004) MrModeltest v2. Program distributed by the author. Evolutionary Biology Centre, Uppsala University. https://www.abc.se/~nylander/mrmodeltest2/mrmodeltest2.html

[B42] Rambaut A Suchard MA Xie D Drummond AJ (2014) Tracer v1.6. Program distributed by the author. http://beast.bio.ed.ac.uk/Tracer

[B43] Reiche KF (1911) Estudios críticos de la Flora de Chile. Anales de la Universidad de Chile 6: 148–159.

[B44] Reimann C Breckle SW (1988) Anatomie und Entwicklung der Blasenhaare von *Chenopodium*-Arten. Flora 180(3–4): 275–288. 10.1016/S0367-2530(17)30323-7

[B45] Scott AJ (1978) A review of the classification of *Chenopodium* L. and related genera (Chenopodiaceae). Botanische Jahrbücher für Systematik. Pflanzengeschichte und Pflanzegeographie 100: 205–220.

[B46] Simón LE (1997) Variations des caractères foliaires chez Chenopodiumsubgen.Ambrosiasect.Adenois (Chenopodiaceae) en Amèrique du Sud: Valeur taxonomique et èvolutive. Adansonia, ser. 3 19(2): 293–320.

[B47] Stamatakis A (2014) RAxML Version 8: A tool for phylogenetic analysis and post-analysis of large phylogenies. Bioinformatics 30: 1312–1313. 10.1093/bioinformatics/btu033 24451623 PMC3998144

[B48] Stöver BC Müller KF (2010) TreeGraph 2: Combining and visualizing evidence from different phylogenetic analyses. BMC Bioinformatics 11(1): 7. 10.1186/1471-2105-11-7 PMC2806359 20051126

[B49] Sukhorukov AP (1999) Eine neue asiatische *Chenopodium*-Art aus der Sektion *Pseudoblitum* Hook. fil. (Chenopodiaceae). Feddes Repertorium 110(7–8): 493–497. 10.1002/fedr.19991100707

[B50] Sukhorukov AP (2006) Zur Systematik und Chorologie der in Russland und benachbarten Staaten (in den Grenzen der ehemaligen UdSSR) vorkommenden *Atriplex*-Arten (Chenopodiaceae). Annalen des Naturhistorischen Museums in Wien 108B: 307–420.

[B51] Sukhorukov AP (2014) The carpology of the Chenopodiaceae with reference to the phylogeny, systematics and diagnostics of its representatives. Grif & Co., Tula. [in Russian with English summary]

[B52] Sukhorukov AP Kushunina MA (2014) Taxonomic revision of Chenopodiaceae in Nepal. Phytotaxa 191(1): 10–44. 10.11646/phytotaxa.191.1.2

[B53] Sukhorukov AP Zhang M (2013) Fruit and seed anatomy of *Chenopodium* and related genera (Chenopodioideae, Chenopodiaceae/Amaranthaceae): Implications for evolution and taxonomy. PLoS One 8(4): e61906. 10.1371/journal.pone.0061906 PMC3633980 23626750

[B54] Sukhorukov AP Uotila P Zhang M Zhang HX Speranskaya AS Krinitsyna AA (2013) New combinations in Asiatic *Oxybasis* (Amaranthaceae s.l.): Evidence from morphological, carpological and molecular data. Phytotaxa 144(1): 1–12. 10.11646/phytotaxa.144.1.1

[B55] Sukhorukov AP Mavrodiev EV Struwig M Nilova MV Dzhalilova KK Balandin SA Erst A Krinitsyna AA (2015a) One-seeded fruits in the core *Caryophyllales*: Their origin and structural diversity. PLoS One 10(2): e0117974. 10.1371/journal.pone.0117974 PMC4339201 25710481

[B56] Sukhorukov AP Zhang M Kushunina M (2015b) A new species of *Dysphania* (Chenopodioideae, Chenopodiaceae) from South-West Tibet and East Himalaya. Phytotaxa 203(2): 138–146. 10.11646/phytotaxa.203.2.3

[B57] Swofford DL (2002) PAUP* Phylogenetic Analysis Using Parsimony (*and other methods). Version 4. Sinauer Associates, Sunderland.

[B58] Taberlet P Gielly L Pautou G Bouvet J (1991) Universal primers for amplification of three non-coding regions of chloroplast DNA. Plant Molecular Biology 17(5): 1105–1109. 10.1007/BF00037152 1932684

[B59] Theodorova TA (2014) Gen. *Chenopodium*, *Blitum* (incl. *Oxybasis*, *Lipandra*, *Chenopodiastrum*). In: Mayevsky PF (Ed.) Flora of the central part of European Russia, Ed.11. KMK Press, Moscow, 91–93.

[B60] Turland NJ Wiersema JH Barrie FR Greuter W Hawksworth DL Herendeen PS Knapp S Kusber WH Li DZ Marhold K May TW McNeill J Monro AM Prado J Price MJ Smith GF (Eds) (2018) International Code of Nomenclature for algae, fungi, and plants (Shenzhen Code): Adopted by the Nineteenth International Botanical Congress Shenzhen, China, July 2017. Regnum Vegetabile 159. Koeltz Botanical Books, Glashütten. 10.12705/Code.2018

[B61] Ulbrich E (1934) Chenopodiaceae. In: Engler A Harms A (Eds) Die Natürlichen Pflanzenfamilien (2nd edn), Vol.16c. Engelmann, Leipzig, 379–584.

[B62] Uotila P (1993) Taxonomic and nomenclatural notes on *Chenopodium* in the Flora Iranica area. Annales Botanici Fennici 30: 189–194.

[B63] Uotila P (1997) *Chenopodium* (s.l.). In: Rechinger KH (Ed.) Flora des Iranischen Hochlandes und der umrahmenden Gebirge, Vol.172. Akademische Druck- und Verlagsanstalt, Graz, 24–59.

[B64] Uotila P (2017) Notes on the morphology and taxonomy of *Chenopodiastrum* (Chenopodiaceae/Amaranthaceae s. lato), with two new combinations, *C.erosum* from Australia and *C.gracilispicum* from China. Annales Botanici Fennici 54(4–6): 345–352. 10.5735/085.054.0616

[B65] Watson S (1871) United States Geological Explorations of the fortieth parallel. Botany, Vol. 5. Government Printing Office, Washington.

[B66] White TJ Bruns T Lee S Taylor J (1990) Amplification and direct sequencing of fungal ribosomal RNA genes for phylogenetics. In: Innis MA Gelfand DH Sninsky JJ White TJ (Eds) PCR Protocols: a guide to methods and applications.Academic Press, New York, 315–322. 10.1016/B978-0-12-372180-8.50042-1

[B67] Wilson PG (1984) Chenopodiaceae. In: George AS (Ed.) Flora of Australia, Vol.4. Australian Government Publishing Service, Canberra, 81–317.

[B68] Zacharias EH Baldwin BG (2010) A molecular phylogeny of North American Atripliceae (Chenopodiaceae), with implications for floral and photosynthetic pathway evolution. Systematic Botany 35(4): 839–857. 10.1600/036364410X539907

[B69] Zhu GL Sanderson SC (2017) Genera and a new evolutionary system of World Chenopodiaceae. Science Press, Beijing.

[B70] Zuloaga FO Morrone O (1999) Catálogo de las plantas de la República Argentina II (Acanthaceae-Euphorbiaceae). Missouri Botanical Garden, St. Louis.

